# Characterization of *Aggregatibacter actinomycetemcomitans* Serotype b Strains with Five Different, Including Two Novel, Leukotoxin Promoter Structures

**DOI:** 10.3390/vaccines8030398

**Published:** 2020-07-20

**Authors:** Rolf Claesson, Huei-Min Chiang, Mark Lindholm, Carola Höglund Åberg, Dorte Haubek, Anders Johansson, Jan Oscarsson

**Affiliations:** 1 Division of Oral Microbiology, Department of Odontology, Umeå University, S-90187 Umeå, Sweden; mark.lindholm@umu.se (M.L.); jan.oscarsson@umu.se (J.O.); 2 Division of Molecular Periodontology, Department of Odontology, Umeå University, S-90187 Umeå, Sweden; HueiMin.Chiang@regionvasterbotten.se (H.-M.C.); carola.hoglund.aberg@umu.se (C.H.Å.); anders.p.johansson@umu.se (A.J.); 3 Section for Paediatric Dentistry, Department of Dentistry and Oral Health, Aarhus University, 8000 Aarhus, Denmark; dorte.haubek@dent.au.dk

**Keywords:** *Aggregatibacter actinomycetemcomitans*, leukotoxicity, leukotoxin promoter types, genotyping

## Abstract

The JP2 genotype of *A. actinomycetemcomitans*, serotype b has attracted much interest during the past three decades due to its close association with periodontitis in young individuals and the enhanced expression of a leukotoxin (LtxA). A typical feature of this genotype is a 530-base pair (bp) deletion in the *ltxCABD* promoter region controlling leukotoxin expression. In the present work, we have characterized serotype b strains with four additional promoter types. Two novel types have been recognized, that is, one with a 230-bp deletion and one with a 172-bp duplication. Moreover, a strain with a 640-bp deletion and three strains with a full-length promoter, including the type strain Y4, were included in the present study. The seven strains were characterized by multi locus sequence typing (MLST) and arbitrarily primed polymerase chain reaction (PCR) and assessed for LtxA production. MLST showed that the strains with the non-JP2-like deletions represented distinct monophyletic groups, whereas the JP2 strain, HK1651, represented a separate branch. LtxA production was high in all three strains with a promoter deletion, whereas the other four strains showed significantly lower levels. It can be concluded that the genetic characterization and determination of LtxA production of *A. actinomycetemcomitans* isolates from individuals with periodontitis can contribute to the identification of novel virulent genotypes of this bacterium.

## 1. Introduction

The toxin-producing bacterium *Aggregatibacter actinomycetemcomitans* is associated with periodontitis in young people [1,2,3,4]. This disease is characterized by a rapid degradation of the tooth-supporting tissues [5]. Since the toxin affects leukocytes of different types, it is called a leukotoxin [6,7]. The affected leukocytes release antibacterial substances and interleukin (IL)-1β, which, in turn, causes activation of the bone-degrading osteoclasts [8]. The leukotoxin is an interesting target for future therapeutic strategies [7,9].

The *ltxCABD* gene operon encoding the leukotoxin (LtxA) is controlled by one promoter, referred to as the leukotoxin promoter [10]. It has a full length of approximately 1106–1112 base pairs (bp), as judged by the whole genome sequences, available in the National Center for Biotechnology (NCBI) (Bethseda, MD, USA) database. On the other hand, some isolates, belonging to the so-called JP2 clone or the JP2 genotype, are characterized by lacking almost half of the full-length promoter, that is, 530 bp [11]. This genotype was initially believed to only be present in individuals of North or West African origin but it has also been detected in individuals of other origins [12,13,14,15,16,17].

The JP2 genotype produces increased amounts of the leukotoxin (LtxA) and strains of this genotype have been demonstrated to be highly leukotoxic [18]. Individuals colonized by this genotype are at increased risk for development of an aggressive form of periodontitis [3,19,20,21]. JP2 genotype strains share a specific gel electrophoresis banding pattern in arbitrarily primed (AP) polymerase chain reaction (PCR), referred to as AP-PCR genotype 1 [22,23]. Non-JP2 AP-PCR genotype 1 strains can also express high leukotoxin levels and are associated with periodontal attachment loss progression [22]. All AP-PCR genotype 1 strains, in contrast to those of the other AP-PCR genotypes analyzed so far, carry the *cagE* gene locus. This gene sequence has been suggested as a suitable diagnostic marker for carriers of highly virulent *A. actinomycetemcomitans* serotype b and which are at increased risk of developing an aggressive form of periodontitis and appears not to be present in any of the other serotypes [23,24].

Whereas AP-PCR typing mirrors genetic variability within bacterial species without catching specific genes, multilocus sequence typing (MLST) is used for the characterization of bacteria in terms of pathogenicity and dissemination by sequencing specific so-called housekeeping genes [22,25]. MLST has been used to follow the worldwide dissemination of the JP2 genotype [25].

In addition to the most common variant with a full-length leukotoxin promoter and the JP2 genotype, two additional promoter variants of *A. actinomycetemcomitans* have been reported, one lacking 640 bp instead of the typical 530 bp and one promoter that carries an 886-bp insertion sequence (IS), referred to as IS1301 [26,27]. In the present work, we reveal the identification and characterization of two additional *ltxCABD* promoter types of *A. actinomycetemcomitans* serotype b, being one, which contains a 172-bp duplication, whereas the other lacks 230 bp and therefore has a deletion. These newly discovered promoter types have been characterized in comparison with the other well-known leukotoxin promoter types regarding AP-PCR and *cagE* genotype, MLST phylogenetic analysis and leukotoxicity.

## 2. Materials and Methods

### 2.1. Routine Procedures for the Quantification, Isolation and Primarily Characterization of A. actinomycetemcomitans from Clinical Samples

Samples from periodontal pockets of patients have been routinely analyzed for the presence of periodontitis–associated bacterial species at the Clinical laboratory at Oral Microbiology, Dental School in Umeå, Sweden, for more than 35 years, according to established methods [13,24].

The samples were collected from periodontal pockets with paper points and sent to the laboratory in an anaerobic transport medium (VMGAIII) [28]. After serial dilution, the samples were spread on blood agar (5% defibrinated horse blood, 5 mg hemin/L, 10 mg Vitamin K/L, Columbia agar base) and on trypticase‒bacitracin‒vancomycin (TBV) plates, an *A. actinomycetemcomitans-*specific medium [29]. After incubation of the blood agar plates in an anaerobic environment at 37 °C for seven days and the TBV plates at 37 °C for three days in an aerobic atmosphere containing 5% CO_2,_ the total number of viable bacteria and the proportion of *A. actinomycetemcomitans* in the samples were calculated. Catalase-positive colonies with star-like inner structure were serotyped and leukotoxin promoter typed as described in Section 2.3. Isolates of the bacterium were collected for further characterization

### 2.2. Bacterial Strains and Growth Conditions Used

*A. actinomycetemcomitans* strains 581-18U, 582-18U and 046-19U were isolated from subgingival plaque using the procedures described above. Five additional serotype b strains were included in this work for comparison purposes. Three of the strains exhibit a full-length leukotoxin promoter—575G was sampled from a Ghanaian cohort of adolescents (1110-bp promoter) [22]. S23A belongs to a collection of oral *A. actinomycetemcomitans* strains previously reported on by Professor Sirkka Asikainen (1110 bp). Y4 (ATCC 43718; 1107 bp) [30,31] is a reference strain. HK1651 [32] is JP2 genotype (530-bp promoter deletion) and 456-13U has a larger, 640-bp promoter deletion [26]. For experimental purposes, the strains were cultivated on blood agar plates, incubated in air supplemented with 5% CO_2_ at 37 °C.

### 2.3. DNA Isolation and Polymerase Chain Reaction-Based Characterization

DNA templates for conventional PCR and quantitative PCR (qPCR) analysis were obtained by boiling a loopful of fresh *A. actinomycetemcomitans* colonies in water. *A. actinomycetemcomitans* genomic DNA to be used in AP-PCR and whole-genome sequencing was isolated using the GenElute™ Bacterial Genomic DNA kit (Sigma-Aldrich, St. Louis, MO, USA), following the manufacturer’s instructions. PCR reaction mixtures were prepared using illustra™ PuReTaq™ Ready-To-Go™PCR beads (GF Healthcare, Little Chalfont, Buckinghamshire, UK), whereas a KAPA SYBR^®^FAST qPCR kit (KAPA Biosystems, Wilmington, MA, USA) was used for qPCR. The AP-PCR type was analyzed as described earlier [13,23], using the random sequence oligonucleotide OPB-3 (5’-AGTCAGCCAC-3’) (Invitrogen, Carlsbad, CA, USA) at 0.4 μmol/L and cycling conditions according to Dogan and collaborators [33]. The strains were grouped into AP-PCR genotype 1, 2 or “other” (i.e., AP-PCR types 3‒11, as defined earlier [13,24]). The *cagE* gene was amplified by qPCR as a 623-bp internal fragment using the primers *cagE*_F2 and *cagE*_R2, as described previously [23]. The primers and PCR cycling conditions used for the serotyping and leukotoxin promoter typing are described elsewhere [34,35,36]. DNA sequencing of PCR fragments, where feasible, was done at Eurofins MWG Operon (Ebersberg, Germany).

### 2.4. Whole Genome Sequencing

Genomic DNA samples from strains 046-19U, 582-18U and Y4 were prepared as described above and sent to MicrobesNG (Birmingham, UK) for whole-genome sequencing. The obtained trimmed reads were annotated with Rapid Annotation using Subsystem Technology (RAST) (rast.nmpdr.org). The genome of the serotype b strain HK1651 (CP007502) was used as the reference. The whole genome sequences of 456-13U and 575G [23] and S23A (GenBank accession GCA_000332955), were also available prior to the present study. The two novel leukotoxin promoter sequence types identified in the present work will be deposited in the European Nucleotide Archive (http://www.ebi.ac.uk/ena).

### 2.5. Multilocus Sequence Typing (MLST)

The gene fragments used in MLST were selected previously, based on the sequences of strain HK1651 [25]. Parts of six genes encoding housekeeping enzymes were used—*recA* (GenBank accession EF142768), encoding the RecA protein; *adk* (EF142164), encoding adenylate kinase; *frdB* (EF142336), encoding fumarate reductase; *atpG* (EF142218), encoding the gamma subunit of ATP synthase F1; *pgi* (EF142653), encoding glucose-6-phosphate isomerase; and *mdh* (EF142568), encoding malate dehydrogenase. In addition, two fragments of the hemoglobin-binding protein pseudogene *hbpA* (EF142408 and EF142489) and one fragment of the transferrin-binding protein pseudogene *tbpA* (EF142817) were selected. Nucleotide sequences were obtained either by sequence analysis of PCR fragments or extracted from whole-genome sequencing data. The oligonucleotides and PCR amplification conditions used have been described previously [25]. A cluster analysis of the seven *A. actinomycetemcomitans* strains used in the present work was conducted, based on concatenated sequences in the order *adk*, *atpG*, *frdB*, *mdh*, *pgi*, *recA*, *hbpA*-*1*, *hbpA*-*2* and *tbpA*. Evolutionary analyses were conducted in MEGA X (Molecular Evolutionary Genetics Analysis) (https://www.megasoftware.net/) [37,38]. Evolutionary history was inferred using the maximum likelihood method and the Tamura‒Nei model, with 1000 bootstrap replicates [39]. A total of 4143 nucleotide positions were used in the final dataset. 

### 2.6. Determination of LtxA Production and Leukotoxic Activity

The leukotoxicity, that is, the capacity of the strains to kill THP1-cells and induce IL-1β release from them, was analyzed. LtxA was extracted from suspensions of 10^10^ bacterial cells/mL (OD_600nm_ = 10.0) by a rocking movement for 1 h in a 0.45% sodium chloride solution in PBS at around 5 °C. The killing assay was based on the reduction of neutral red uptake in LtxA-exposed THP-1 cells stained with this dye. The concentration of THP-1 cells (10^6^) was determined in a hemocytometer before they were added to a 96 welled cell culture plate. Cellular neutral red uptake was quantified by a spectrophotometer and expressed as a percent of a plane control, as described previously [40]. The assessment of the median lethal dose (LD_50_), the dose that kills 50% of the target cells, was determined in leukotoxin-exposed THP-1 cultures. The target cells were exposed for 2 h to leukotoxic extracts from different concentration of the various *A. actinomycetemcomitans* isolates or to purified LtxA. The concentration (multiplicity of infection (MOI)) that reduced viability in the THP-1 cultures with 50% determined the LD_50_ value. LtxA-induced release of IL-1β from the target cells was measured with an enzyme-linked immunosorbent assay (ELISA) kit in accordance to the manufacturer’s protocol (RnD Systems, Abingdon, UK).

For the determination of the LtxA production with checkerboard (CB), a Western blot-based method was used. A suspension of 2 × 10^9^ bacterial cells/mL (OD_600nm_ = 2.0) of plate-cultivated isolates were disintegrated by boiling for 5 min together with 1% SDS (1:1). The samples were loaded in a Miniblotter device (Miniblotter 45MN, Interchim, Montlucon, France) with a nitrocellulose (NC) membrane. The NC membrane was treated stepwise with rabbit–anti LtxA [8] and goat-anti rabbit–HRP (DAKO/AS, Roskilde, Denmark). For visualizing LtxA specific bands, Super Signal was used (Pierce, Rockford, IL, USA). The banding intensity of the different strains was quantified in relation to the corresponding band of a reference strain (HK1651) using a ChemiDoc^TM^ XRS + System (BioRad, Solna, Sweden). Data were expressed as a percent (%) of the banding intensity for the JP2 genotype, HK651. 

### 2.7. Ethical Considerations

Ethical clearance for the present study was obtained from the Noguchi Memorial Institute for Medical Research, University of Ghana (IRB 000 1276) and from the local ethical committee of Umeå University, Sweden (Dnr 2010-188-31M). 

### 2.8. Statistical Calculations 

The statistical significance of the leukotoxicity data was calculated with ANOVA and a post-hoc test with Bonferroni correction to determine the *p*-values. The 95% confidence interval (*p* ≤ 0.05) was used for the indication of significant differences from the JP2 genotype of *A. actinomycetemcomitans* (HK1651).

## 3. Results

### 3.1. Identification of Two Novel Leukotoxin Promoter Types in A. actinomycetemcomitans Serotype b

**Figure 1:**
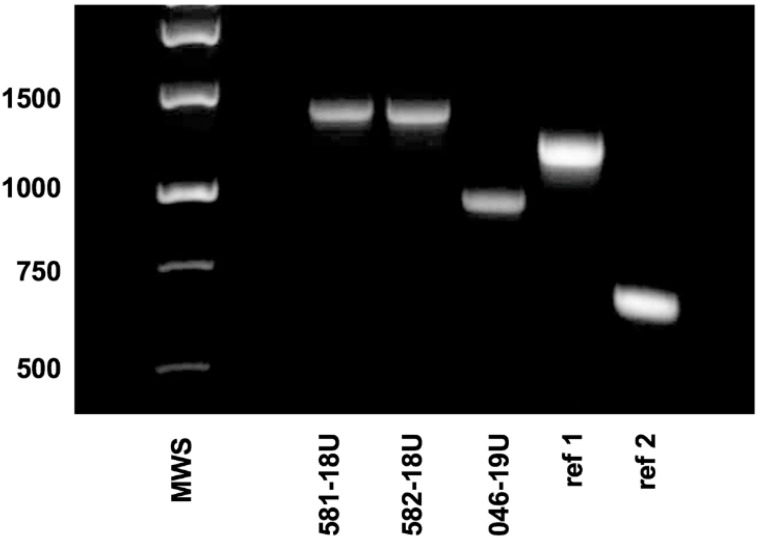
Polymerase chain reaction (PCR) analysis of the leukotoxin promoter region of *A. actinomycetemcomitans* strains 581-18U, 582-18U and 046-19U. As controls, DNA from a strain with a complete, full-length leukotoxin promoter (ref 1) and from a strain with a JP2 genotype promoter (530-bp deletion; ref 2) was amplified. Sizes (bp) of selected bands in the DNA molecular weight standard (MWS) are indicated.

We recently identified two novel types of leukotoxin promoter of *A. actinomycetemcomitans* in our clinical laboratory of the Dental School at Umeå, Sweden. One was detected in two periodontal plaque samples collected from a 21-year-old female patient of Ethiopian descent and with severe periodontal lesions located on the first molar. These isolates were denoted 581-18U and 582-18U (Figure 1). The samples were collected from deep pockets with high proportions of *A. actinomycetemcomitans* (Table 1). The second novel promoter type was detected in a periodontal plaque sample containing low proportions of *A. actinomycetemcomitans* (isolate denoted 046-19U) (Table 1 and Figure 1). It was collected from a 22-year-old female periodontitis patient from Syria. 

**Figure 2:**
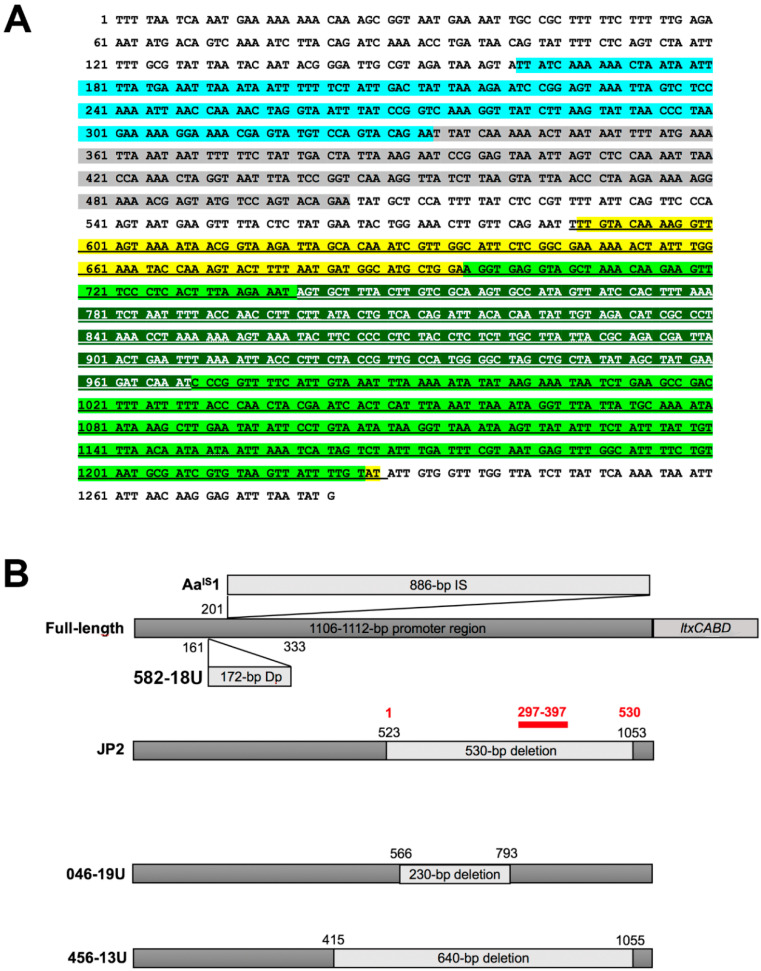
The five different *ltxCABD* promoter structures of the *A. actinomycetemcomitans* strains characterized in the present study. (**A**) The DNA sequence of the promoter region of *A. actinomycetemcomitans* 582-18 is shown, which contains 1110 bp—starting from the first nucleotide after the stop codon of the gene *glyA* and including the start codon of *hlyC* [41]—and the 172-bp duplication (highlighted in blue and gray). Deletions have been highlighted in the additional indicated colors. The 640-bp deletion of strain 456-13U (bp 415–1055) [26] (underlined and highlighted in yellow), the JP2 genotype 530-bp deletion (bp 523–1053) [41] (light green) and the 230-bp deletion of isolate 046-19 (bp 566–796) (dark green and white letters). (**B**) Schematic illustration of the different promoter structures, including the one of strain Aa^IS^1, containing an 886-bp IS-element [42] and that of 046-19U containing a 172-bp duplication (Dp). The site within the JP2 promoter deletion (bp 297–397) that has been suggested to be bound by a repressor is indicated as a red bar [43].

The isolates were confirmed to be of serotype b according to PCR analysis (data not shown); however, deviant patterns were observed when the leukotoxin promoter regions of the isolates were amplified by PCR (Figure 1). DNA sequencing revealed that 172 bp of the promoter region was duplicated in strains 581-18U and 582-18U, that is, bp position 161–333 in the promoter. On the other hand, strain 046-19U was found to have a 230-bp deletion at bp position 566–796 (Figure 2A,B). The two novel promoter type strains were subjected to genotypic and phenotypic characterization in comparison with five additional strains, as representatives of the other previously recognized promoter types. The type strain Y4 was subject to whole-genome sequencing as the sequence available in the NCBI database appears to be that of a serotype a strain.

### 3.2. Genotypic Characterization of the Two Novel Promoter Type Strains

**Figure 3:**
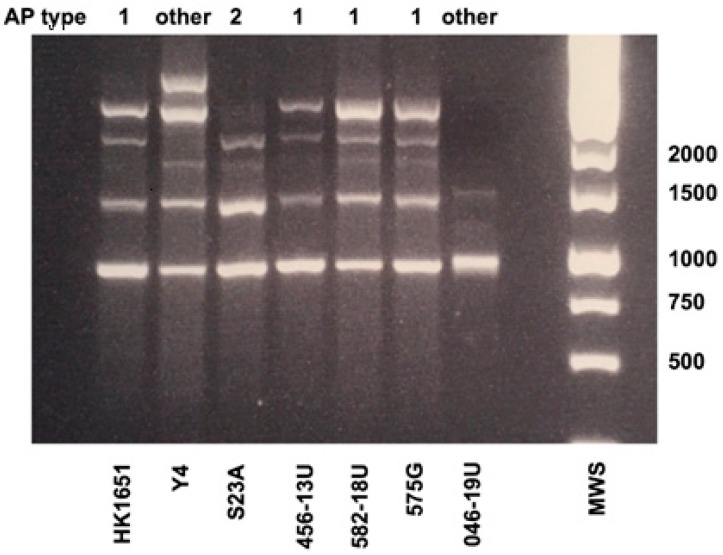
Arbitrarily primed (AP)-PCR genotyping of the seven *A. actinomycetemcomitans* serotype b strains. Distinct banding patterns distinguish AP-PCR types 1, 2 and “other” (AP-PCR types 3‒11, as defined previously [13]). Sizes (bp) of selected bands in the DNA molecular weight standard (MWS) are indicated.

AP-PCR characterization of the isolates showed that the strain with the duplication (582-18) was an AP-PCR type 1, whereas the novel deletion type, strain 046-19U, was found to be an AP-PCR “other” type (Figure 3). 

**Figure 4:**
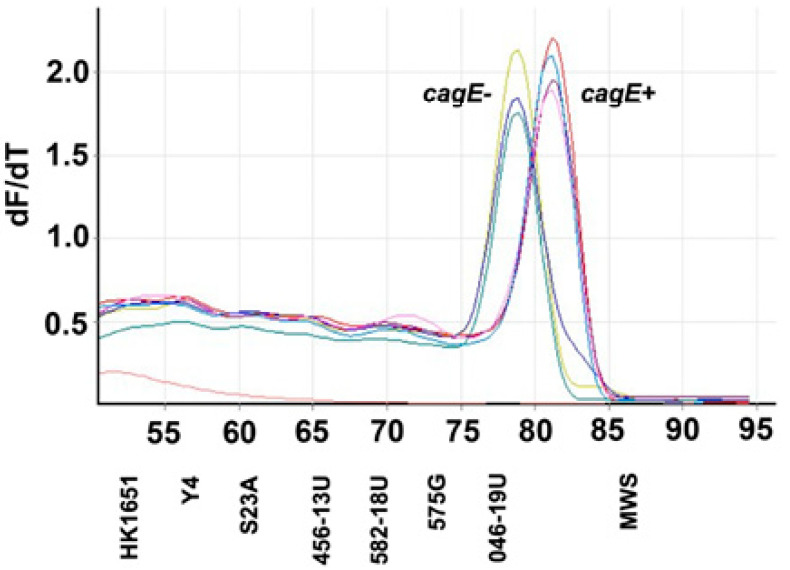
Carriage of *cagE* by *A. actinomycetemcomitans* strains with different leukotoxin promoters. Shown are melt curves obtained in quantitative PCR, using the oligonucleotide primers *cagE*_F2 and *cagE*_R2, which generate a single, distinct amplicon with a melting temperature (Tm) of 81 °C for *cagE*-positive strains (HK1651, 456-13U, 575G and 582-18U). For *cagE*-negative strains (S23A, 046-19 and Y4) an amplicon with a lower Tm was obtained, consistent with previous studies [23]. Graphs show rates of change of fluorescence relative to temperature (dF/dT).

Consistent with being of AP-PCR type 1, isolate 582-18U, according to qPCR, carried the *cagE* gene locus, in contrast to 046-19U, which was found to be *cagE*-negative (Figure 4). 

### 3.3. Multilocus Sequence Typing and Phylogenetic Tree

To analyze the evolutionary distances between the seven strains, representing five leukotoxin promoter types, MLST was performed next, using nine gene fragments, as described in Section 2. Based on the single-nucleotide polymorphisms (SNPs; *n* = 8) observed in these gene fragments, six MLST sequence types could be identified, denoted 1‒6. No variation among the strains were observed in the *adk*, *mdh* and *recA* gene fragments. Strain Y4 was the only one to have a SNP in the *frdB* fragment and the *hbpA1*-fragment was shorter in strains 046-19U and Y4, containing only nucleotide positions 370‒439 (Table 2). 

**Figure 5:**
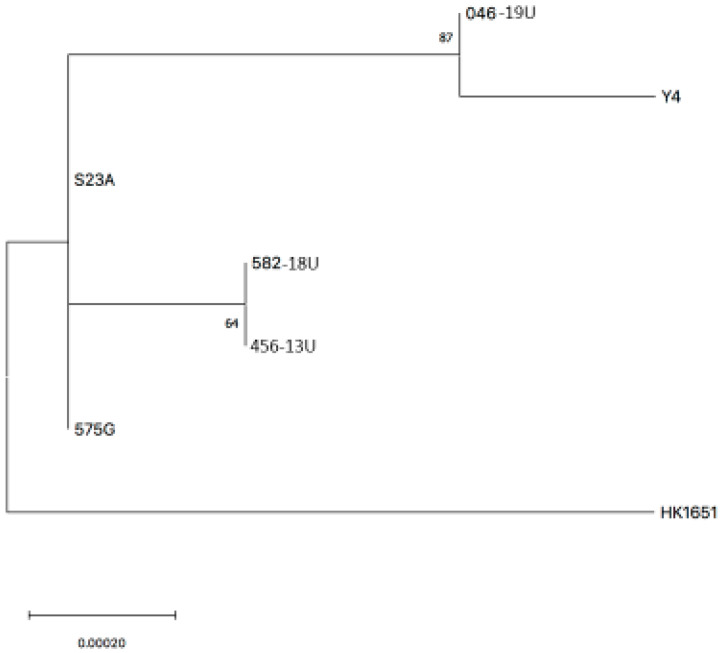
Phylogenetic relationships among the seven *A. actinomycetemcomitans* serotype b strains, representing five different *ltxCABD* promoter types, based on multilocus sequence typing (MLST) analysis, as described in Section 2. The tree with the highest log likelihood is shown. The percentages of trees in which the associated strains clustered together is shown next to the branches. The tree is drawn to scale, with branch lengths measured in the number of substitutions per site.

Cluster analysis, using the nine concatenated sequences, showed that there was a rather close relationship between the strains. Strains 046-19U and Y4 and 456-13U and 582-18U, respectively, represented distinct monophyletic groups, whereas the JP2 strain, HK1651, represented a separate branch (Figure 5). 

### 3.4. The Two Novel Promoter Type Strains Exhibit Different Leukotoxicity

**Figure 6:**
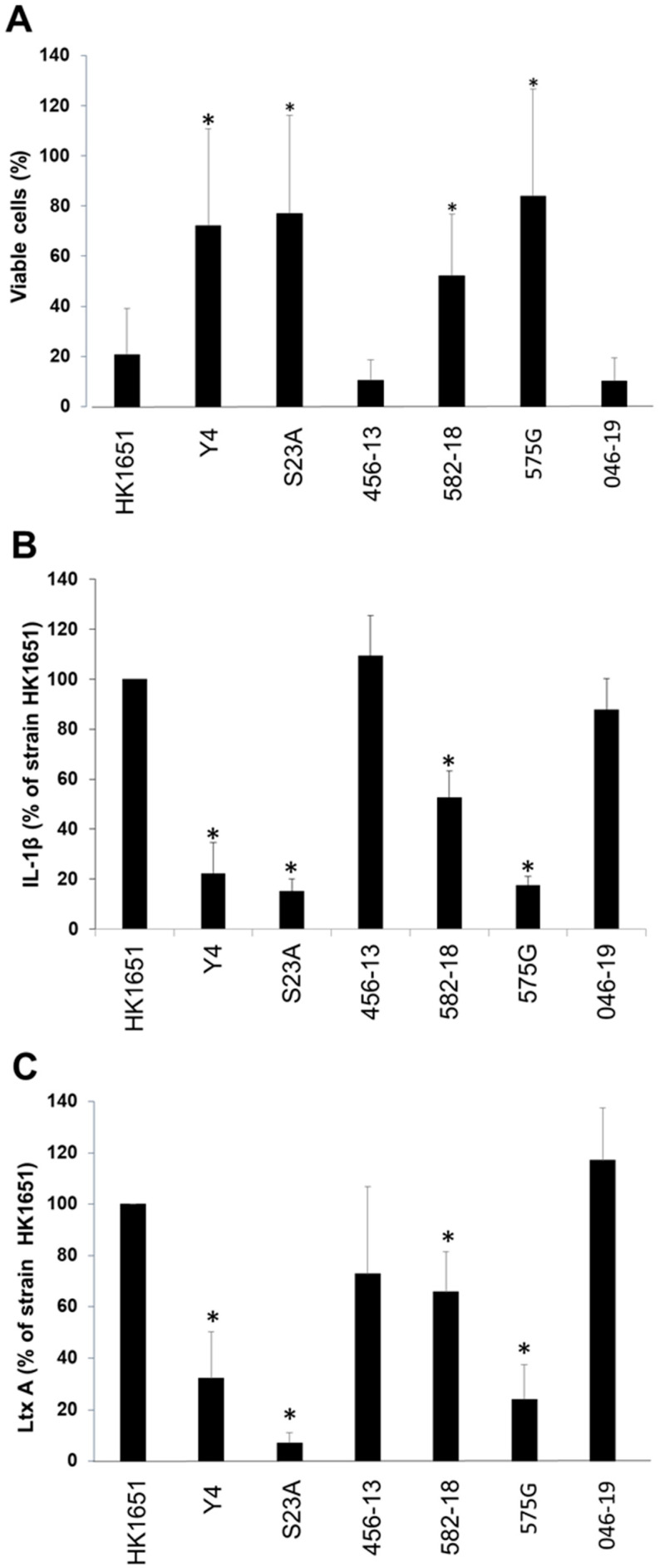
Leukotoxicity of *A. actinomycetemcomitans* strains with different leukotoxin promoters and significant differences (*p* < 0.05) from the JP2 genotype are indicated (*). (**A**) The leukotoxicity was quantified as killing of THP-1 cells by leukotoxin extracted from the bacterial cells. Cell viability was monitored as quantification of neutral red uptake in the exposed THP-1 cells. MOI 100, that is, extracts from 10^8^ bacterial cells was incubated with 10^6^ THP-1 cells for 2 h. Mean ± SD of five experiments is shown. (**B**) The leukotoxicity was quantified as leukotoxin-induced release of IL-1β from THP-1 cells. Released IL-1β was quantified by ELISA-based technique. MOI 10, that is, extracts from 10^7^ bacterial cells were incubated with 10^6^ THP-1 cells for 2 h. Mean ± SD of three experiment is shown. (**C**) The production of leukotoxin was quantified by a checkerboard-based technique, including the usage of leukotoxin antibodies. Mean ± SD of five different experiments is shown.

Next, leukotoxic activity and leukotoxin production were analyzed by three different methods, comparing seven strains with different promoter types, including 582-18U and 046-19U. According to the method based on leukotoxicity, isolates 456-13U and 046-19U were considered highly leukotoxic, that is, showing a similar leukotoxicity to HK 1651 (JP2 type) (Figure 6A). The LD_50_ values for the killing capacity of each isolate or of purified LtxA, indicated a significantly higher leukotoxicity of the three isolates with promoter deletions, HK1651, 456-13U, 046-19U and 582-18U, compared to the three other isolates (Table 3). 

The three highly leukotoxic *A. actinomycetemcomitans* isolates also induced a higher secretion of IL-1β from the exposed THP-1 cells than the four other isolates (Figure 6B). According to the immune detection method (CB), based on production of the LtxA protein, strains HK1651, 456-13U and 046-19U produced high amounts of the toxin (Figure 6C), whereas the LtxA protein levels detected in the other four isolates (Y4, S23A, 582-18U and 575G) were significantly lower. Collectively, based on the results of all three methods, strains Y4, S23A, 575G and 582-18U were considered weakly leukotoxic, with a significantly higher LtxA production in HK1651, 456-13U and 046-19U, carrying the different promoter deletions (Figure 6A–C).

## 4. Discussion

In the present work, we have revealed the identification and characterization of two novel leukotoxin promoter types of *A. actinomycetemcomitans.* Thus, to the best of our knowledge, there are now five recognized promoter structures. 

*A. actinomycetemcomitans* strains having a full-length (≈1106–1112 bp) promoter structure are generally considered weakly leukotoxic. However, a subset of serotype b strains with full-length promoters can exhibit high leukotoxicity. Properties in common for them are that they share the same AP-PCR type, that is, AP-PCR type 1 [22] and that they carry the *cagE* gene locus [23,24]. *A. actinomycetemcomitans* strains with specific genetic rearrangements within the promoter, both deletions and insertions, have been reported to be highly leukotoxic [11,26,42]. In the present study we contribute with two novel leukotoxin promoter types, one with a 172-bp duplication and one with a 230-bp deletion. As one phenotypic characterization of these novel leukotoxin promoter type strains, the LtxA expression was determined and compared with that of the other five strains. 

For studying LtxA expression, various methods have been used over several decades [11,22,44,45,46]. In the present study, methods based on both leukotoxicity and on assessment of the leukotoxin protein levels (checkerboard) revealed similar results, that is, the leukotoxin expression was significantly higher for the JP2 genotype strain, HK1651, the 640-bp deletion type (456-13U) and the 230-bp deletion type (046-19U) as compared to strains Y4, S23A and 575G. However, the reason why the checkerboard method revealed a higher LtxA expression for strain 582-18U than the leukotoxicity-based methods is not known. 

The generally low production of LtxA by *A. actinomycetemcomitans* with a complete leukotoxin promoter has been suggested to be due to the fact that a transcriptional repressor, hitherto unknown, can bind to a region in the promoter (bp 523–1053), which is not present in JP2 genotype strains due to their 530-bp deletion [41]. Indeed, recently, it was reported that the specific region of the promoter that is crucial for the binding of this putative repressor is 100 bp, present within the 530-bp region (bp 297–397), that is absent in the JP2 genotype strains [43]. As expected, the 230 bp-deletion in strain 046-19 was found to be located within the same 530-bp region. It would be of interest to identify and characterize suppressor(s) that target this region.

However, interestingly, by being deprived of bp 44-264 the promoter of strain 046-19U actually contains the region where the potential repressor might bind. This suggests that other regions of the promoter may also be targeted by regulatory protein(s). This would be consistent with observations of the *A. actinomycetemcomitans* strain *Aa*^IS^1, which has an 886-bp IS1301 insertion in the *ltxCABD* promoter [42]. That promoter is otherwise intact and the reason *Aa*^IS^1 produces enhanced amounts of LtxA has been proposed to be that IS1301 displaces an upstream negative cis-acting DNA sequence [47]. To the best of our knowledge, strain 582-18U represents the first finding of a duplication within the leukotoxin promoter. Moreover, interestingly, this could also be the first example of a promoter rearrangement that has not resulted in an apparent increase in leukotoxicity, despite its close proximity to the site for the IS1301 insertion in strain Aa^IS^1. The reason for this discrepancy is not known. 

Like the leukotoxic activity, the AP-PCR pattern varied among the tested strains. Among them, four are AP-PCR type 1 (HK1651, 456-13U, 582-18U and 575G). This AP-PCR type has been found to be more associated with enhanced leukotoxicity than the other AP-PCR-types [22,23]. Among the seven strains studied in the present work, five followed this pattern, whereas for 046-19U and 575G the opposite was observed. The AP-PCR gel electrophoresis pattern of 046-19U is not often detected among serotype b strains of *A. actinomycetemcomitans*, which indicates that the 230-bp leukotoxin promoter type is interestingly divergent in more than one aspect. However, like the other non-AP-PCR 1 types, it does not carry *cagE*. 

When the genetic variability of the strains was studied by MLST, six sequence types were recognized based on the identification of eight SNPs. Although the numbers of SNPs differed substantially between the strains, their genetic distance was relatively low, as shown in the phylogenetic tree. Among the four genetic subgroups identified by the MLST in the present work, the JP2 genotype strain, HK1651 was represented in a separate branch. MLST analysis of 66 JP2 strains has revealed that they are closely related genetically [25]. Interestingly, despite the two strains (456-13U and 046-19U) having leukotoxin promoter deletions partly overlapping those of JP2 strains, they displayed a limited genetic relationship to HK1651. Instead, a close genetic relationship was observed between strains 456-13U and 582-18U. Intriguingly, both strains originated from Ethiopia/Eritrea. A further relationship to consider is that of strain 046-19U, which originates from Syria and the type strain Y4, which together represented a monophyletic branch in the MLST analysis. They were the only strains found to carry an SNP (A > G) at position 24 within the *tpbA* gene fragment. In addition, Y4 carries an SNP (T > C) at position 131 within the *frdB* gene. None of these SNPs could be observed in any of the 66 JP2 strains studied earlier [25].

**Figure 7:**
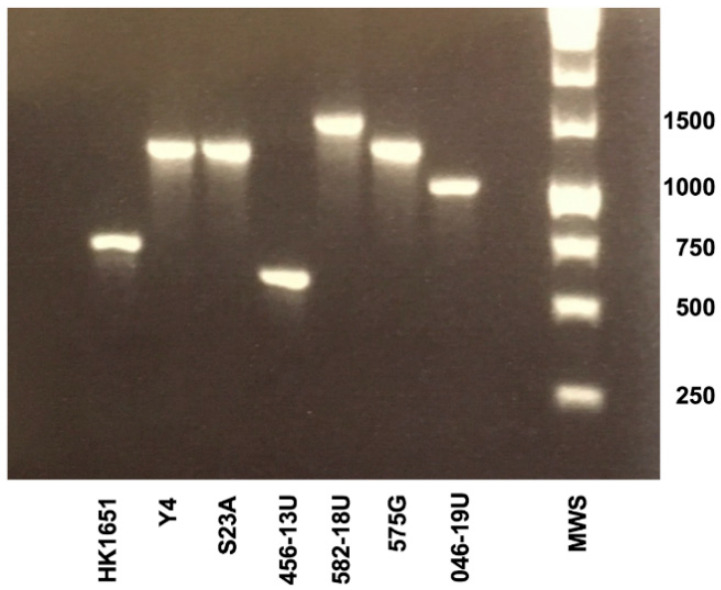
PCR analysis of the leukotoxin promoter region of all *A. actinomycetemcomitans* (*Aa*) strains compared in the present study, representing five different *ltxCABD* promoter types. Sizes (bp) of selected bands in the DNA molecular weight marker (MWS) are indicated.

Among the five different leukotoxin promoter types of *A. actinomycetemcomitans* that we have focused on in the present study, the one with the complete promoter type (≈1106–1112 bp) is without doubt the most common (Figure 7). 

The JP2 genotype is mainly detected among individuals of African origin [18]. Individuals of non-African origin carrying the JP2 genotype do exist but are not frequently reported [12,13,14,15,16,17]. The widespread geographic locations of these observations raise the question of the clonality for the JP2 genotype, which most likely will require further genome sequencing data to elucidate. The 886-bp IS1301 variant has been observed twice, both times in Asia [42,48]. Interestingly, both the JP2-like promoter type (530-bp deletion) and the 886-bp insertion promoter type have been identified in serotype c in one study [48]. Otherwise, all hitherto known deletions and insertions in the *ltxCABD* promoter have been restricted to serotype b. The reason for this is not known but may possibly reflect the fact that the promoter is not assessed to the same extent regarding the other serotypes. 

Indeed, the serotype b 640-bp and 230-bp deletion-variants and the 172-bp duplication variant were detected in our laboratory after analyzing 5072 periodontal samples from 2223 individuals during 20 years (2000–2019). Among these individuals, 693 carried *A. actinomycetemcomitans.* While each novel leukotoxin promoter was carried by one individual, the carriage of the JP2 genotype was detected among 30 of the 693 individuals. Carriers of the JP2 genotype are at increased risk for development of an aggressive form of periodontitis due to production of enhanced levels of the leukotoxin. Here, we show that other leukotoxin promoters types could be highly leukotoxic, indicating that not only the JP2 genotype should be in focus when periodontitis risk patients are to be identified and treated. However, for supporting the hypothesis that the novel leukotoxin promoter types here described have pathogenic capacity additional findings are required. 

Overall, it seems that the prevalence of *A. actinomycetemcomitans* with deleted or extended leukotoxin promoters is very low. There are reasons to believe that the prevalence could be substantially underestimated due to the absence of sampling and leukotoxin promoter typing, especially regarding the other serotypes. This also means that access to valuable laboratory diagnostics parameters for treatment of periodontal diseases is limited, as well as for studies of the geographic dissemination of *A. actinomycetemcomitans* with various leukotoxin promoter structure.

## 5. Conclusions

The identification of two additional leukotoxin promoter types of *Aggregatibacter actinomycetemcomitans* has extended the concept of leukotoxin promoter structure versus leukotoxicity, genetic diversity and geographic dissemination of this bacterium. 

## Figures and Tables

**Table 1:** The proportion of *A. actinomycetemcomitans* (*Aa*) in the subgingival plaque samples was quantified by cultivation of the samples on blood agar plates (total viable count (TVC)) and on *Aa*–selective plates (TBV agar).

Sample	TVC per Sample, Millions	*Aa* per Sample, Millions	*Aa* % of TVC	Tooth Site	PPD * mm
581-18U	13	2.9	22	26	8
582-18U	13	6.6	51	46	8
046-19U	32	0.02	0.05	14	11

* Periodontal probing pocket depth.

**Table 2:** Polymorphic sites for six *A. actinomycetemcomitans* strains with various leukotoxin promoter types, as compared to the JP2 strain HK1651, characterized by a 530-bp deletion. Nucleotides in genes/gene fragments sequenced.

	adk	atpG	frdB	mdh	pgi	recA	hbpA1 ^2^	hbpA2	hbpA2	tpbA	tpbA	MLST Type ^3^
		231 ^1^	131		509		44	6	214	24	56	
456-13U		G > C			C > T		T > G	T > G	A > G		A > -	6
046-19U		G > C						T > G	A > G	A > G	A > -	4
Y4		G > C	T > C					T > G	A > G	A > G	A > -	5
582-18U		G > C			C > T		T > G	T > G	A > G		A > -	6
S23A		G > C										2
575G		G > C						T > G	A > G		A > -	3

^1^ Nucleotide position (bp) in the corresponding gene of HK1651; ^2^ the *hbpA1* fragment of 046-19 and Y4 contains only nucleotide positions 370‒439; ^3^ HK1651 represents MLST type 1.

**Table 3:** The number of *A. actinomycetemcomitans* (*Aa*) cells/THP-1 cell (MOI) that killed 50% of the target cells after 2 h exposure (LD_50_). The viability of the exposed THP-1 cells was examined with quantitative neutral red uptake. Mean ± SD of five experiments are shown. *p*-value vs. HK1651 is indicated.

*Aa* Strain	LD_50_ MOI	SD	*p*-Value
HK1651	35	±40	-
Y4	544	±377	0.036
S23A	676	±400	0.002
456-13U	11	±10	1
582-18U	142	±99	1
575G	458	±435	0.178
046-19U	18	±12	1
LtxA *	33	±19	-

* LD_50_ for LtxA is expressed in ng/mL.
